# Development and validation of dust exposure prevention questionnaire for cardiovascular patients based on the health belief model

**DOI:** 10.1186/s12889-020-09871-3

**Published:** 2020-11-25

**Authors:** Farzaneh Noroozi, Kumars Eisapareh, Alireza Bahadori, Leila Ghahremani, Rosanna Cousins, Hamidreza Mokarami

**Affiliations:** 1 Department of Health Education and Promotion, School of Health, Shiraz University of Medical Sciences, Shiraz, Iran; 2 Applied Meteorological Research Center, Bushehr, Iran; 3 Department of Psychology, Liverpool Hope University, Liverpool, UK; 4 Department of Ergonomics, School of Health, Shiraz University of Medical Sciences, PO Box 71645-111, Shiraz, Iran

**Keywords:** Cardiovascular disease, Dust, Health belief model, Psychometric properties

## Abstract

**Background:**

Many cardiovascular patients suffer from respiratory failure. Environmental conditions can exacerbate symptomatology. It is necessary to prevent exposure to dust by taking educational steps to identify and modify patient behavior. This study aimed to develop and validate a dust exposure behavior questionnaire based on the Health Belief Model.

**Methods:**

A mixture of qualitative and quantitative methods was employed to design and develop the desired tool. Qualitative methods were used to identify the preventive behaviors needed by cardiovascular patients at risk of dust exposure using the opinions of two expert panels and a literature review. The quantitative phase of the research was performed to evaluate the psychometric properties of the research tool. The research population comprised 417 people with cardiovascular disease referred to a heart hospital in Bushehr, Iran in 2018. Consenting participants entered the study through consecutive sampling.

**Results:**

The final version of the questionnaire included 27 items across six domains, namely perceived susceptibility, perceived barriers, perceived severity, perceived benefits, cues to action, and self-efficacy. The mean values of the content validity ratio and content validity index were 0.93 and 0.9, respectively. In addition, all items had a good correlation with the total score of their parent domain (*P* < 0.01). The model fit was initially unsuitable, according to the related indices. Hence, to achieve a better model fit, the model was improved by releasing some parameters based on the modifications suggested by the AMOS software. The modified model featured an acceptable fit (χ2/df = 2.2, *P* < 0.001). Cronbach’s alpha coefficients also confirmed appropriate reliability for all six domains.

**Conclusion:**

The Dust Exposure Prevention questionnaire has desirable psychometric properties and appropriate validity to determine the behavioral factors involved in harm from dust exposure among cardiovascular disease patients. This marks an effective step toward evaluating the factors effective in preventing complications related to dust exposure among such patients.

## Background

**Fig. 1:**
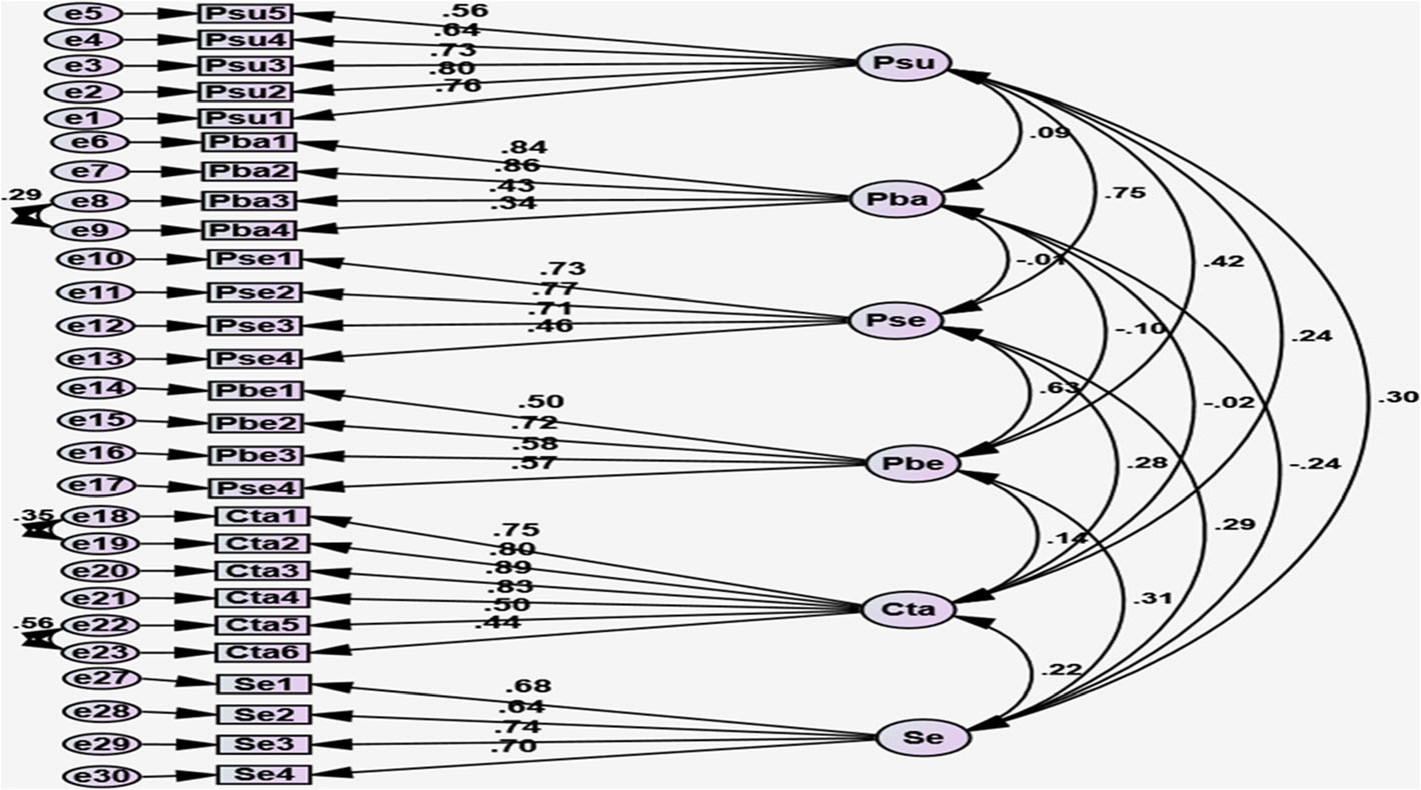
Confirmatory factor analysis factor for the Dust Exposure Prevention questionnaire for heart patients. Abbreviations: Psu **=** Perceived susceptibility; Pba = Perceived barriers; Pse = Perceived severity; Pbe = Perceived benefits; Cta = Cue to action; Se = Self-efficacy

Cardiovascular diseases (CVDs) represent the leading source (48%) of mortality and they are the fifth-largest cause of disability worldwide [1, 2]. Cardiovascular diseases account for most non-communicable disease deaths, i.e., approximately 17.9 million annually [3]. Despite some inroads into reducing the number of cardiovascular deaths since 1979, there has not been any improvement in the rate of hospitalization among patients under 55 years of age [4]. Statistics indicate that 72% of deaths in Iran are caused by non-communicable diseases, and that CVD accounts for almost half of this figure [1]. The mortality rate of CVD in Iran is about 150,000 individuals per year [5].

Environmental risk factors (dust, toxic substances, pollution, etc.) comprise one of the five risk factors for CVD that can exert both direct and indirect effects on health [6], and many CVD patients suffer from respiratory failure [7]. Particles sized ≤2.5 μm adversely affect human health and augment the rate of mortality due to respiratory failure related to CVD and lung cancer. Long-term exposure to a 10 μg/M^3^ concentration of pollutants raises the mortality rate by 6%, and the occurrence of CVD and lung cancer by 12 and 14%, respectively. Moreover, there is significant evidence that both long-term and short-term exposure to fine particles in the form of air pollution have a negative impact on cardiovascular health (see Pope and Dockery [8] for a full discussion). Related to this, is a robust study conducted to investigate the relationship between particle-induced air pollution and the admission of patients with myocardial infarction in seven states of the USA in 2006. The findings indicated that if the concentration of particles sized ≤10 μm increased by 10 μg/M^3^, then the rate of patient admission on the same day would increase by 10% [9]. There is also evidence of a direct relationship between exposure to high levels of air pollution (walking in dusty weather) and the occurrence of acute coronary ischemia [10]. A study examining 775 CVD patient’s understanding of risk factors associated with their disease and rehabilitation indicated relatively poor knowledge of the causes of CVD generally, and low levels of appreciation of the contribution of environmental factors to CVD. Nevertheless, more than half of the men and a quarter of the women in that sample recognized that their behaviors were an important contributory cause of their illness [11]. Given all the points mentioned, it can be appreciated that to reduce the prevalence of CVDs and support rehabilitation it is necessary to prevent exposure to dust by taking educational steps to modify behavior among those with CVDs.

Human behavior is a reflection of various factors, and knowledge is a prerequisite for changing behaviors [12]. A low level of knowledge and poor performance in avoiding exposure to risk factors of CVDs affect the exposure, incidence, and exacerbation of these diseases [13]. Confirmed models, such as the Health Belief Model (HBM) [14], can help to systematically identify factors that can support behavioral modifications, making it easier to achieve the desired improvements. The HBM [14] is one of the most widely used models for explaining changes in behavior at the level of the individual [15, 16]. This theoretical model is underpinned by the notion that particular beliefs influence the way a person behaves. It applies to health behaviors with the potential to reduce risk of developing a disease, as well as behaviors that impact upon an existing disease. The HBM is an expectancy-value model. The emphasis is on two attributes of an individual’s interpretation of a health problem and their own behavior. These are threat perception and behavior evaluation. Threat perception is conceptualized on the basis of two beliefs – a person’s perceived susceptibility to a disease or illness, and their perceived severity of the consequences of that disease or illness. Behavior evaluation is also conceptualized on two beliefs – perceived benefits of adopting a particular health behavior, and perceived barriers to adopting the required health behavior to achieve benefit. The model also proposes there are a diverse range of triggers, or ‘cues to action’ that can activate a change in behavior, and that a person’s willingness to be concerned about their health has an influence on supporting behavior change. Thus there are six constructs that make up the HBM [12].

Baghani et al., conducted a study to examine the role of health beliefs in preventive behaviors of individuals at risk of CVDs. Their findings strongly suggest that preventive behaviors can be enhanced by raising awareness about the perceived benefits and minimizing the perceived barriers [17]. Studies have also shown that public perceptions play a role in identifying and predicting environmental damage to human health. For instance, an investigation conducted in southern Sweden demonstrated how perceived contamination and perceived health risks played a more important role in being aware of and predicting damage caused by air pollutant odors than direct exposure to the odors [18]. Other research reports have also reported a significant relationship between patients’ attitudes concerning CVD and related preventive behaviors [19].

Modifying personal beliefs about health risk factors can promote a healthy lifestyle [20]. Studies on the primary prevention of stroke through lifestyle modifications have revealed that 47% of strokes in women and 35% of strokes in men can be due to a high-risk lifestyle [21]. It has also been asserted that CVD is a chronic condition with environmental causes, and that behavioral modifications can substantially reduce the risk of CVD [22].

A review of the literature suggested that the HBM has not previously been used to modify exposure to dust in patients with CVD. Furthermore, previous questionnaires based on the HBM for CVD patients are not suitable for this purpose because these questionnaires are either combinations of different structures of other questionnaires [23] or have disadvantages such as a lack of content and structure validity (i.e., weak psychometric properties), failure to use all of the health belief model structures, and content irrelevance [24].

Due to the high prevalence of dust in recent years and the impact of this phenomenon on CVD, especially in the Middle East and in Iran, it is necessary to conduct further research in this area to determine the effective factors for intervention. This requires the use of valid tools for the prevention of dust-exposure among CVD patients. The use of validated questionnaires is an important step in generalizing and completing the research implementation cycle. Thus, given the importance of the subject, the need for a questionnaire with good psychometric properties became irrefutable. Hence, the aim of present study was to develop and validate a dust prevention questionnaire based on the HBM.

## Methodology

### Study design

The current research was conducted in the city of Bushehr, Iran in 2018. A mixture of qualitative and quantitative methods was used to design and develop the desired tool. The qualitative phase of the research was performed to identify the preventive behaviors needed by cardiovascular patients when exposed to dust. The items were developed using an expert panel and a literature review. The quantitative phase was designed to evaluate the psychometric properties of the research tool. The research design was approved by the Scientific and Ethical Committee of Bushehr University of Medical Sciences (IR.BPUMS.REC.1395.62).

### Design of the questionnaire

The research tool was designed in four steps based on the Waltz Tool Design Method [25]:

- *Step (1):* Dust-related complications for CVD patients were conceptualized. Definitions were based on the six dimensions of the HBM and were derived through a literature review and the input of a panel of experts. To review the texts, valid databases including SID, IranMedex, Scopus, and PubMed were searched using the following keywords: ‘*heart disease’, ‘cardiopulmonary problems’, ‘dust’,* and *‘health belief model’*. The panel of ten experts included cardiovascular, health education, and environmental health professionals, all of whom were university professors (Bushehr University of Medical Science) and familiar with the HBM. The experts were invited to participate in the study based on their known expertise and willingness to support research. All provided written consent in response to the invitation letter.
  The objective of the concept explanation stage was to present a comprehensive definition of dust-related complications for cardiac patients based on the six dimensions of the HBM.
- *Step (2):* The purpose of this step was to determine whether the tool would be applicable for the particular setting. Arriving at an operational definition for each of the six domains of the HBM was an essential part of this step [26].
- *Step (3):* The tool items were formulated. An initial item pool was developed based on the definitions and the text of the six dimensions of the HBM for cardiac patients [23, 24]. The items were created and reviewed in three sessions by the panel of experts that participated in Step 1.
- *Step (4):* The psychometric characteristics of the tool were examined. One of the most important characteristics that indicate the applicability of a tool is its reliability and validity [26]. In this step, the following measures were taken:

#### Face validity and content validity

Content validity was examined using both qualitative and quantitative methods. Content validity refers to the extent to which the tool items are related to the studied content or conceptual dimensions [27]. The ten professors that made up the qualitative panel of experts were asked to examine the grammar, wording, and item allocation of the Dust Exposure Prevention tool being developed. If the given principles were not observed, they would be asked to suggest a correction for the items. In addition, in order to eliminate any possibility of ambiguity and promote easy understanding of the items, the views of ten cardiac patients not included in the quantitative phase were obtained and their considered corrections were applied to the items.

Content validity index (CVI) and content validity ratio (CVR) were used to evaluate the content validity. To support the process of making these calculations a new expert panel consisting of four cardiovascular professors, four health education professors and two environmental health professors was formed (using the same process as described above). According to Lawshe [28] the CVR determines the necessity of an item based on a three-point Likert scale (necessary, useful but not necessary, or not necessary). The minimal value of the CVR for each item was calculated using the formula below:

$$ \mathrm{CVR}=\frac{\mathrm{Number}\ \mathrm{of}\ \mathrm{essential}\ \mathrm{answers}\ \mathrm{for}\ \mathrm{each}\ \mathrm{item}-\frac{\mathrm{Total}\ \mathrm{number}\ \mathrm{of}\ \mathrm{respondents}}{2}}{\frac{\mathrm{Total}\ \mathrm{number}\ \mathrm{of}\ \mathrm{respondents}}{2}} $$

As ten experts were used to calculate CVRs, following Lawshe’s Table [28], items with a ratio above 0.62 would have an acceptable level of significance (*P* ≤ 0.05) and thus be retained.

The CVI is a measure for determining the appropriateness, clarity, ambiguity, and relevance of questionnaire items to research objectives from the experts’ points of view [29]. For this purpose, a four-point Likert scale was used to evaluate the opinions of the second expert panel of professors with regards to the relevance (not relevant, fairly relevant, relevant, or very relevant), clarity (not clear, fairly clear, clear, or very clear) and simplicity (not simple, fairly simple, simple, or very simple) of each item.

$$ \mathrm{CVI}=\frac{\mathrm{The}\ \mathrm{number}\ \mathrm{of}\ \mathrm{experts}\ \mathrm{giving}\ \mathrm{scores}\ 3\ \mathrm{or}\ 4\ \mathrm{to}\ \mathrm{the}\ \mathrm{item}}{\mathrm{Total}\ \mathrm{number}\ \mathrm{of}\ \mathrm{experts}} $$

Items that did not have an appropriate index score were excluded.

The questionnaire was also evaluated by the ten cardiac patients. They were asked to appraise the importance of the remaining items to provide an Impact Score for each item. The patients rated the importance of each item using a five-point Likert scale (very important, important, moderately important, slightly important, or not important, scored in order from five to one). Then, the Impact Score for each item was calculated using the following formula: “Impact Score = Frequency (%) x Importance”. “Frequency” in the formula referred to the number of patients that rated an item 4 or 5, while “Importance” was the mean score of the item on the 1–5 rating scale. If the Impact Score of an item was higher than 1.5, then the item was identified as appropriate and was retained for subsequent analyses [30].

#### Construct validity

Construct validity examines the relationship between a measurement tool and its theoretical background. In other words, construct validity assesses the extent to which a measurement tool reflects theorems: the better this reflection, the higher the construct validity. In order to use factor analysis, there must be a correlation between the desired variables. When the matrix is ​​significant, all correlation coefficients will equal to zero [31]. Confirmatory factor analysis (CFA), with the likelihood maximal method at the level of the covariance matrix, was used to evaluate the construct validity using the HBM and to identify the tool domains. This method was used because CFA is a statistical method that tests hypothetical models, and this is not possible using conventional multivariate tools such as exploratory factor analysis (EFA).

### Participants and questionnaire test procedure

The research population included people with CVDs referred to a heart hospital in Bushehr, Iran in 2018. Inclusion criteria were a diagnosis of CVD, aged between 18 and 75 years, and the ability to read and write. The exclusion criterion was a lack of exposure to dust in the previous year (self-reported). Using the consecutive sampling method, 571 patients who met the inclusion criteria were provided with a participant information sheet and invited to join the study. Four hundred ninety-eight people (87.2%) agreed to be participants. Patient interviews and data collection were done by a health education and promotion specialist. Before administering the anonymized questionnaire, the objectives and methodology of the study were explained to the patients and their written informed consent was obtained. In this way, the patients were assured that participation in the study was voluntary and that the data would only be analyzed collectively. Necessary explanations were provided by the questioner to low-literacy patients requesting support, and their responses carefully recorded.

At the end of the questionnaire data collection step from the initial 498 participants, 46 questionnaires submitted by participants who reported that they had not being exposed to dust in the previous year, and 35 questionnaires with incomplete information (i.e. more than 20% missing data) were excluded from the analysis. This yielded a final sample of 417 participants (73% of potential sample). It should be noted that for the structural analysis, the sample size was between 4 and 10 times more than the number of items in the questionnaire (30). Hence, the number of participants was suitable for evaluating the questionnaire’s psychometric properties.

To evaluate the fitness of the CFA model, the chi-square index was used first. Values smaller than the mentioned index indicate a better fit of the model. However, as this index is sensitive to large sample sizes, the researchers did not rely on this index and calculated the chi-square to degree of freedom ratio to ensure statistical significance. It has been recommended that the chi-square to degree of freedom ratio should be less than three for a model to be accepted [24]. Other indices used to evaluate the CFA model were the comparative fit index (CFI), incremental fit index (IFI), root mean square error of approximation (RMSEA), goodness of fit index (GFI), and the adjusted goodness of fit index (AGFI). The CFI, IFI, GFI, and AGFI each take a value between zero and one. The closer the values are to one, the more appropriate the model is [32]. Additionally, RMSEA values less than 0.08 are considered to be appropriate and those less than 0.05 are regarded as a good fit [31, 33]. Finally, GFI and AGFI values higher than 0.8 and 0.9, respectively, and CFI values higher than 0.9 are considered to be appropriate [27, 31]. The data were analyzed using AMOS software, version 23 (IBM, USA).

#### Reliability

To determine the internal stability of the questionnaire and each of its domains, the internal consistency method was used. In this regard, the use of Cronbach’s alpha coefficient is one of the most common methods based on the internal consistency of the scales within a questionnaire [34]. The data were analyzed using SPSS software, version 23 (IBM, USA).

## Results

Two hundred twenty-four participants (53.5%) were male and 193 (46.5%) were female. Most of the participants had high school education (*n* = 182, 43.5%) or elementary education (*n* = 162, 39%), and only 73 (17.5%) possessed academic degrees. The duration of cardiac disease was less than 1 year in almost half of the patients (46%), while more than half (54%) had undergone surgery.

Based on the HBM, the operational definitions of the six domains that comprised the new Dust Exposure Prevention questionnaire were as follows:

- Perceived susceptibility: One’s belief that they should not be exposed to dust due to the impact on cardiac disease.
- Perceived severity: One’s belief that exposure to dust may exacerbate their cardiac disease and may even lead to hospitalization.
- Perceived barriers: One’s belief that there are difficulties for them in terms of taking the recommended preventive measures.
- Perceived benefits: One’s belief that they will benefit from performing preventive behaviors.
- Perceived self-efficacy: One’s belief in their capabilities in performing preventive behaviors.
- Cues to action: The presence of internal (individual signs and symptoms) or external (external persons or warning signs) factors that act as a guide for performing preventive behaviors.

Questionnaire items were designed based on the articles, books, and questionnaires as well as the panel of experts in regard to the consequences of dust exposure for cardiac patients based on the constructs of the HBM. The initial version included 55 items. After examination by the panel of experts, some items were altered or excluded because they either failed to represent the relevant dimension (five items) or they were unable to convey a clear expression of the intended point (20 items). Following this elimination process, 30 items were selected from the item pool and grouped under the six domains of the HBM. Of these 30 items, only four items were related to previous research tools, and the rest of the items were developed by the panel of experts specifically for this questionnaire. The questionnaire domains were each scored using a five-point Likert scale:

1. Perceived severity: five items answered according to likelihood (very high, high, moderate, low, or very low). Item scores ranged from one for very low to five for very high. Accordingly, the minimum score was five and the maximum score was 25.
2. Perceived barriers: four items similarly answered very high - very low. The minimum score was four, and the maximum score was 20.
3. Perceived susceptibility: four items answered according to agreement with the statement (strongly agree, agree, neutral, disagree, or strongly disagree). Item scores ranged from one for strongly disagree to five for strongly agree. Thus, the minimum and maximum scores were four and 20, respectively.
4. Perceived benefits: four items similarly answered strongly agree - strongly disagree. Scores were a minimum of four and a maximum of 20.
5. Cues to action: nine items, each answered according to frequency (always, most often, sometimes, rarely, or never). The scores ranged from one for never to five for always. Accordingly, the minimum score of this dimension was nine and its maximum score was 45.
6. Self-efficacy: four questions similarly answered according to frequency (always, most often, sometimes, rarely, or never). The minimum score was four and the maximum score was 20.

### Validity

Based on the qualitative content validity and according to the recommendations of the experts as well as the ambiguities raised by the patients, written changes were applied to the questionnaire items though no items were deleted. Mean CVR and CVI values were 0.93 and 0.9, respectively (Table 1).

**Table 1:** Content Validity Ratio (CVR) and Content Validity Index (CVI) of Items

Domain	Item	CVR	CVI	Final items
**Perceived sensitivity**	1. To what extent does dust in the air exacerbate CVDs?	0.9	0.8	✓
	2. To what extent does dust in the air exacerbate a heart attack?	1	1	✓
	3. To what extent does dust in the air trigger a heart attack?	0.9	0.8	✓
	4. To what extent does dust in the air increase the mortality rate of CVDs?	1	0.9	✓
	5. To what extent does dust in the air reduce the effectiveness of treatments?	0.9	0.9	✓
**Perceived barriers**	6. It is difficult for me to use a mask during times of air pollution.	0.9	0.8	✓
	7. It is difficult for me to use a filtered mask during times of air pollution.	1	1	✓
	8. Staying home on dusty days is boring for me.	1	0.9	✓
	9. On dusty days, despite worsening symptoms, visiting a doctor or a medical center is difficult for me.	0.9	0.7	✓
**Perceived severity**	10. Airborne dust can aggravate CVDs.	0.9	1	✓
	11. Existence of dust in the air can trigger dangerous heart attacks in CVDs.	0.8	0.9	✓
	12. Existence of dust in the air can increase the mortality rate of CVDs.	0.9	0.9	✓
	13. Existence of dust in the air can reduce the effectiveness of treatments.	1	0.8	✓
**Perceived benefits**	14. Wearing a filter mask on high dust days can reduce the risk of complications.	1	0.8	✓
	15. Staying in the house on a very dusty day is good for maintaining health.	1	1	✓
	16. Immediate referral to a doctor can prevent heart problems if symptoms occur on dusty days.	0.9	0.9	✓
	17. Paying attention to air pollution announcements is beneficial to protect the health of the community.	0.7	0.9	✓
**Cues to action**	18. My doctor advises me to use a mask when the air is dusty.	1	0.9	✓
	19. My doctor advises me not to go out of the house when the weather is dusty.	1	0.8	✓
	20. Health center staff advise me to use a mask in dusty weather.	0.9	1	✓
	21. Health center staff advise me not to go out of the house when the weather is dusty.	1	0.9	✓
	22. My family and friends advise me to use a mask when the weather is dusty.	1	1	✓
	23. My family and friends advise me not to go out of the house when the weather is dusty.	1	0.9	✓
	24. I pay attention to the mass media warnings about using a mask when the weather is dusty.	0.9	1	–
	25. I pay attention to the mass media warnings about not going out of the house when the weather is dusty.	1	0.9	–
	26. I have friends who inform me when the weather is dusty.	0.8	1	–
**Self-efficacy**	27. I can still wear a mask even when it is difficult to use in times of air pollution.	1	0.9	✓
	28. Even if I have work to do during times of air pollution, I can stay at home.	0.8	1	✓
	29. I am able to pay more attention to my symptoms when the air is heavily polluted.	0.9	0.7	✓
	30. On days when I can’t go out of the house due to air pollution, I can entertain myself at home.	1	0.8	✓

In the face validity determination stage, the results obtained by calculating the item impact indices showed that the impact scores of all items were greater than 1.5. Thus, all items were appropriate for determining content validity. In this step, all items achieved the minimum score of construct validity. Therefore, all 30 items were assessed for their construct validity using CFA. Before implementing the factor analysis, the correlation between the score of each item and the scores of all items in each domain was examined. The results revealed that all of the items had a good correlation with the total score of their related domains fit (*P* < 0.01) (see Table 2). In other words, these items had the necessary discriminatory power to measure the desired domains.

**Table 2:** Corrected Item-Total Correlation and Cronbach’s Alpha of Items

Domain	Item	Mean (SD)	Corrected Item-Total Correlation	Cronbach’s Alpha if Item Deleted	Cronbach’s Alpha
Perceived susceptibility	Psu1	3.86 (1.0)	.660	.774	.825
	Psu2	3.97 (.99)	.683	.768	
	Psu3	3.75 (1.0)	.651	.777	
	Psu4	3.56 (1.0)	.585	.796	
	Psu5	3.46 (1.0)	.506	.819	
Perceived barriers	Pba1	3.09 (1.2)	.601	.617	.727
	Pba2	3.20 (1.3)	.613	.607	
	Pba3	3.22 (1.3)	.470	.694	
	Pba4	3.24 (1.2)	.394	.734	
Perceived severity	Pse1	4.34 (.76)	.530	.688	.757
	Pse2	4.10 (.81)	.655	.617	
	Pse3	3.95 (.83)	.599	.646	
	Pse4	3.61 (1.0)	.407	.777	
Perceived benefits	Pbe1	4.16 (.77)	.368	.664	.678
	Pbe2	4.34 (.71)	.557	.547	
	Pbe3	4.16 (.84)	.460	.607	
	Pbe4	4.25 (.77)	.453	.610	
Cues to action	Cta1	3.80 (1.3)	.646	.815	.842
	Cta2	3.72 (1.3)	.652	.815	
	Cta3	3.69 (1.3)	.678	.812	
	Cta4	3.47 (1.4)	.638	.816	
	Cta5	3.68 (1.2)	.599	.822	
	Cta6	3.69 (1.1)	.595	.822	
	Cta7	3.66 (1.1)	.404	.840	
	Cta8	3.64 (1.1)	.463	.835	
	Cta9	2.95 (1.3)	.331	.850	
	Se1	3.51 (1.1)	.578	.736	
	Se2	3.42 (1.1)	.574	.738	
	Se3	3.80 (.97)	.610	.722	
	Se4	3.72 (1.1)	.598	.724	

The results of CFA on the default model showed that the factor loadings of the 30 items in all six domains were significant. However, the factor loads of three items in the Cues to action domain, including item 7, “I pay attention to the mass media warnings regarding the use of masks when the air is dusty” (*β* = 0.28), item 8, “I pay attention to the mass media warnings regarding not leaving the house when the weather is dusty” (*β* = 0.29), and item 9, “I have friends who inform me when the air is contaminated” (*β* = 0.25), were low. Therefore, these three items were deleted. This provided a tool comprising 27 items. Furthermore, the results showed that the model fit indices were initially unsuitable. Hence, to fully fit the model to the data, we attempted to improve the model by releasing some parameters based on the modifications suggested by the AMOS software. The CFA path chart, after the release of these parameters with standardized factor loadings of the items, is illustrated in Fig. 1. The results of the fit indices of the initial and modified models are presented in Table 3. Based on the standardized fit indices and coefficients and the critical rate index (Table 4), the modified model had an acceptable fit to the data (χ^2^/*df* = 2.2, *P* < 0.001).

**Table 3:** Fit indices of the CFA of the questionnaire

Model fit index	Default model	Modified model
Chi-Square/Degrees of Freedom Ratio *(*χ^*2*^*/df)*	1446 / 390 = 3.708 *P* < 0.001	674 / 306 = 2.20 *P* < 0.001
Goodness-Of-Fit Index (GFI)	.80	.90
Adjusted Goodness-Of-Fit Index (AGFI)	.75	.90
Incremental fit index (IFI)	.79	.92
Comparative Fit Index (CFI)	.77	.92
Root Mean Square Error of Approximation (RMSEA)	.08	.05

χ ^2^/*df* = 2.2, *P* < 0.001; GFI = 0.90; AGFI = 0.90; IFI = 0.92; CFI = 0.92; RMSEA = 0.05

**Table 4:** Items’ loading factor and critical rates of dimensions of questionnaires

Domain	Item	Standardized Regression Weight	Critical Rate	***P***
Perceived susceptibility	Psu1	.757	10.79	< .001
	Psu2	.796	11.06	< .001
	Psu3	.732	10.59	< .001
	Psu4	.639	9.76	< .001
	Psu5	.556	–	–
Perceived barriers	Pba1	.840	–	–
	Pba2	.857	11.92	< .001
	Pba3	.432	8.20	< .001
	Pba4	.336	6.36	< .001
Perceived severity	Pse1	.726	8.56	< .001
	Pse2	.767	8.72	< .001
	Pse3	.711	8.49	< .001
	Pse4	.460	–	–
Perceived benefits	Pbe1	.499	–	–
	Pbe2	.718	8.17	< .001
	Pbe3	.583	7.57	< .001
	Pbe4	.574	7.51	< .001
Cue to action	Cta1	.746	–	–
	Cta2	.801	20.20	< .001
	Cta3	.895	17.55	< .001
	Cta4	.826	16.62	< .001
	Cta5	.496	9.76	< .001
	Cta6	.441	8.65	< .001
Self-efficacy	Se1	.677	–	–
	Se2	.639	10.62	< .001
	Se3	.739	11.69	< .001
	Se4	.705	11.38	< .001

### Reliability

Cronbach’s alpha coefficients showed that the reliability of all six domains was appropriate. The results also revealed an appropriate correlation between the items and the total score of each domain. The Cronbach’s alpha, mean score, corrected item-total correlation, and Cronbach’s alpha of the deleted items for each domain are provided in Table 2.

## Discussion

This study aimed to design and develop a tool for evaluating preventive behaviors related to dust exposure in cardiac patients based on the Health Belief Model [14]. The designed questionnaire included a total of 27 items divided across the six domains of perceived susceptibility (five items), perceived barriers (four items), perceived severity (four items), perceived benefits (four items), cues to action (six items), and self-efficacy (four items). The validity and reliability, including face and content validity, construct validity, and internal consistency, indicated that the psychometric properties of the final questionnaire were shown to appropriate. As no tool has previously been designed regarding the prevention of dust exposure among heart disease patients, the present instrument should be considered as an innovation. The content validity of new tools must be measured and reported if they are going to be used for research [35]. In the present study, the CVR and CVI were measured, and the values of both indices were good, rendering all items suitable for their purpose. The content validity of the total items of the final questionnaire also suggested that the instrument is valid.

Validity refers to the purpose of designing a tool. A valid test is that it possesses the necessary adequacy to measure the subject of study. It seems that less attention has been paid to this aspect of validity of tools in many studies, while much attention has been paid to the research methodology or data analysis. In other words, the provision of sufficient information on the validity and reliability of the tools has been overlooked in some studies conducted in the area of tool design based on the HBM. However, this issue was taken into account in the present study, comprising one of the strengths of our research.

In order to examine the validity of the tool, we did not consider the opinions of health education professionals to be sufficient and also made use of the viewpoints and suggestions of cardiologists and environmental health professionals. In the validation step, a range of valuable opinions were collected. This means that the validity of the tool was evaluated from various angles. The results showed that the six different domains of the questionnaire all had high internal consistency, and values in line with the acceptable values presented in statistical texts [36]. The findings of credible international articles were also referred to in confirmation of the results of the current study [37]. In this study, CFA was employed to examine the construct validity of the instrument. Before performing the principal component analysis, the appropriateness of the data for factor analysis was assessed. The study aimed to evaluate the validity of the research tool based on the psychometric process with relevant details, as far as is possible, in order to provide appropriate evidence to ensure the tool’s validity.

The present study, similar to other studies, had some limitations. The small literature in this field was one of the limitations. Moreover, as research on health education has mainly focused on behavior and the nature of the behavior is complex, items are recommended to be added to evaluate the individuals’ behaviors in the prevention of dust exposure. Furthermore, this study was conducted with the support of patients of only one hospital in Bushehr, Iran. A lack of actual measurements and predictions of behavior change was another limitation, though this was beyond the scope of the current research and represents the next step for future research.

## Conclusions and practical implications

The present study attempted to design a valid tool that assists in preventing dust-related complications among cardiac patients by gathering relevant behavioral data. It also aimed to assure researchers regarding the appropriateness of the designed tool by providing sufficient information about its validity and reliability. The results showed that the designed questionnaire possessed desirable psychometric properties to determine the factors involved in preventing dust-related complications among heart patients. This tool may also be adapted for other similar environmental threats. Given the importance of preventing dust exposure in reducing the prevalence and progression of CVDs, the formulation of this questionnaire represents an effective step toward evaluating the effective factors in preventing complications related to dust exposure among patients with CVDs. This questionnaire can be used as a self-administered tool to assess patient attitudes about preventive behaviors pertaining to dust exposure. Researchers can also use this tool to evaluate related intervention programs.

## Data Availability

The datasets used and/or analyzed during the current study are available from the corresponding author on reasonable request.

## Abbreviations

AGFI: Adjusted Goodness of Fit Index
CFA: Confirmatory Factor Analysis
CFI: Comparative Fit Index
CVD: Cardiovascular diseases
CVI: Content Validity Index
CVR: Content Validity Ratio
GFI: Goodness of Fit Index
HBM: Health Belief Model
IFI: Incremental Fit Index
RMSEA: Root Mean Square Error of Approximation

## References

[1] Alwan A. Global Status Report on Noncommunicable Diseases 2010. Geneva: World Health Organization; 2011. ISBN 9789241564229.

[2] Imanipour M, Bassampour S, Haghani H (2008). Relationship between preventive behaviors and knowledge regarding cardiovascular diseases. Hayat..

[3] World Health Organization. Noncommunicable diseases. Fact Sheet 1 June 2018. [Available from: https://www.who.int/news-room/fact-sheets/detail/noncommunicable-diseases.

[4] Wilmot KA, O’Flaherty M, Capewell S, Ford ES, Vaccarino V (2015). Coronary heart disease mortality declines in the United States from 1979 through 2011: evidence for stagnation in young adults, especially women. Circulation..

[5] Khazaei H, Komasi S, Zakiei A, Rezaei M, Hatamian P, Jashnpoor M (2018). Design and standardization of tools for assessing the perceived heart risk and heart health literacy in Iran. Ann Card Anaesth.

[6] Komasi S, Saeidi M (2015). Aging is an important cause for a lack of understanding of the main risk factor in cardiac rehabilitation patients. Thrita..

[7] Ping RA (1996). Estimating latent variable interactions and quadratics: the state of this art. J Manag.

[8] Pope CA, Dockery DW (2006). Health effects of fine particulate air pollution: lines that connect. J Air Wast Manag.

[9] Wellenius GA, Schwartz J, Mittleman MA (2006). Particulate air pollution and hospital admissions for congestive heart failure in seven United States cities. Am J Cardiol.

[10] Hassing H, Twickler TB, Kastelein J, Cramer M, Cassee F (2009). Air pollution as noxious environmental factor in the development of cardiovascular disease. Neth J Med.

[11] Saeidi M, Komasi S, Soroush A, Zakiei A, Shakeri J (2014). Gender differences in patients’ beliefs about biological, environmental, behavioral, and psychological risk factors in a cardiac rehabilitation program. J Cardio-Thoracic Med.

[12] Glanz K, Rimer BK, Viswanath K. Health behavior: theory, research, and practice. San Francisco: Wiley; 2015. ISBN 9781118628980.

[13] Tavassoli E, Hasanzadeh A, Ghiasvand R, Tol A, Shojaezadeh D (2010). Effect of health education based on the Health Belief Model on improving nutritional behavior aiming at preventing cardiovascular disease among housewives in Isfahan. J Sch Public Health Inst Public Health Res.

[14] Rosenstock IM, Stretcher VJ, Becker MH (1988). Social learning theory and the health belief model. Health Educ Q.

[15] Green J, Cross R, Woodall J, Tones K (2019). Health promotion: planning and strategies.

[16] Jones CJ, Smith H, Llewellyn C (2014). Evaluating the effectiveness of health belief model interventions in improving adherence: a systematic review. Health Psychol Rev.

[17] Baghianimoghadam MH, Mirzaei M, Rahimdel T (2012). Role of health beliefs in preventive behaviors of individuals at risk of cardiovascular diseases. Health Sys Res.

[18] Claeson A-S, Lidén E, Nordin M, Nordin S (2013). The role of perceived pollution and health risk perception in annoyance and health symptoms: a population-based study of odorous air pollution. Int Arch Occup Environ Health.

[19] Hirani SP, Newman SP (2005). Patients’ beliefs about their cardiovascular disease. Heart..

[20] Soroush A, Komasi S, Saeidi M, Heydarpour B, Carrozzino D, Fulcheri M (2017). Coronary artery bypass graft patients’ perception about the risk factors of illness: educational necessities of second prevention. Ann Card Anaesth.

[21] Chiuve SE, Rexrode KM, Spiegelman D, Logroscino G, Manson JE, Rimm EB (2008). Primary prevention of stroke by healthy lifestyle. Circulation..

[22] Bhatnagar A (2017). Environmental determinants of cardiovascular disease. Circulation Res.

[23] Horwood H, Williams MJ, Mandic S (2015). Examining motivations and barriers for attending maintenance community-based cardiac rehabilitation using the health-belief model. Heart Lung Circ.

[24] Shojaei S, Farhadloo R, Aein A, Vahedian M (2016). Effects of the health belief model (HBM)-based educational program on the nutritional knowledge and behaviors of CABG patients. J Tehran Heart Cent.

[25] Waltz CF, Strickland OL, Lenz ER. Measurement in nursing and health research. 4th ed. New York: Springer Publishing Company; 2009. ISBN9780826105073.

[26] Salehi P, Pouladi S, Yazdankhahfard M, Mirzaei K (2018). Designing and psychometric assessment of the questionnaire for artificial airway patients’ satisfaction with nurse's non-verbal communication during nursing cares. Iran J Nurs.

[27] Hsu I-Y, Su T-S, Kao C-S, Shu Y-L, Lin P-R, Tseng J-M (2012). Analysis of business safety performance by structural equation models. Saf Sci.

[28] Lawshe CH (1975). A quantitative approach to content validity. Personnel Psychol.

[29] Polit DF, Beck CT, Owen SV (2007). Is the CVI an acceptable indicator of content validity? Appraisal and recommendation. Res Nurs Health.

[30] Carpenter S (2018). Ten steps in scale development and reporting: a guide for researchers. Commun Methods Meas.

[31] Maasoumi R, Mokarami H, Nazifi M, Stallones L, Taban A, Yazdani Aval M (2017). Psychometric properties of the Persian translation of the sexual quality of life–male questionnaire. Am J Mens Health.

[32] Kline RB (2015). Principles and practice of structural equation modeling: Guilford publications.

[33] Cangur S, Ercan I (2015). Comparison of model fit indices used in structural equation modeling under multivariate normality. J Modern App Stat Meth.

[34] Munro BH (2005). Statistical methods for health care research.

[35] Polit DF, Beck CT (2006). The content validity index: are you sure you know what’s being reported? Critique and recommendations. Res Nurs Health.

[36] Boardley ID, Kavussanu M (2007). Development and validation of the moral disengagement in sport scale. J Sport Exercise Psychol.

[37] Craig CL, Marshall AL, Sjöström M, Bauman AE, Booth ML, Ainsworth BE (2003). International physical activity questionnaire: 12-country reliability and validity. Med Sci Sports Exerc.

